# The Lancet Weight Determines Wheal Diameter in Response to Skin Prick Testing with Histamine

**DOI:** 10.1371/journal.pone.0156211

**Published:** 2016-05-23

**Authors:** Hjalte H. Andersen, Anna Charlotte Lundgaard, Anne S. Petersen, Lise E. Hauberg, Neha Sharma, Sofie D. Hansen, Jesper Elberling, Lars Arendt-Nielsen

**Affiliations:** 1 SMI^®^, Department of Health Science and Technology, Faculty of Medicine, Aalborg University, Aalborg, Denmark; 2 The Allergy Clinic, Copenhagen University Hospital, Gentofte, Copenhagen, Denmark; Michigan State University, UNITED STATES

## Abstract

**Background:**

Skin prick test (SPT) is a common test for diagnosing immunoglobulin E-mediated allergies. In clinical routine, technicalities, human errors or patient-related biases, occasionally results in suboptimal diagnosis of sensitization.

**Objective:**

Although not previously assessed qualitatively, lancet weight is hypothesized to be important when performing SPT to minimize the frequency of false positives, false negatives, and unwanted discomfort.

**Methods:**

Accurate weight-controlled SPT was performed on the volar forearms and backs of 20 healthy subjects. Four predetermined lancet weights were applied (25 g, 85 g, 135 g and 265 g) using two positive control histamine solutions (1 mg/mL and 10 mg/mL) and one negative control (saline). A total of 400 SPTs were conducted. The outcome parameters were: wheal size, neurogenic inflammation (measured by superficial blood perfusion), frequency of bleeding, and the lancet provoked pain response.

**Results:**

The mean wheal diameter increased significantly as higher weights were applied to the SPT lancet, e.g. from 3.2 ± 0.28 mm at 25 g to 5.4 ± 1.7 mm at 265 g (p<0.01). Similarly, the frequency of bleeding, the provoked pain, and the neurogenic inflammatory response increased significantly. At 265 g saline evoked two wheal responses (/160 pricks) below 3 mm.

**Conclusion and clinical relevance:**

The applied weight of the lancet during the SPT-procedure is an important factor. Higher lancet weights precipitate significantly larger wheal reactions with potential diagnostic implications. This warrants additional research of the optimal lancet weight in relation to SPT-guidelines to improve the specificity and sensitivity of the procedure.

## Introduction

Skin prick test (SPT) is the most commonly used test for diagnosing immunoglobulin E (IgE)-mediated sensitization in asthma, allergic rhinitis and food allergy [1,2]. Collectively, these conditions are, to a varying extend, affecting an estimated 25% of the entire western population [3–7], thus warranting continuous optimization of related diagnostic tools and therapies. In SPT reactions to introduced allergens, in sensitized individuals, IgE cross-links with the allergens and binds to the receptor FcεRI on mast cells which in turn causes mast cell degranulation with release of different inflammatory mediators such as histamine and mast cell proteases [2,8]. Histamine stimulates the histamine-1 (H_1_) receptor on the endothelial cells and histamine-2 (H_2_) receptors on smooth muscle resulting in a plasma extravasatory reaction known as a *wheal* in the immediate surroundings of the penetration site [9]. Neurogenic inflammation is a result of a secondary release of vasoactive substances from antidromically activated peptidergic C-fibers [10–12]. The presence and extent of these local reactions (almost exclusively the wheal) are widely used as the day-to-day diagnostic biomarkers for IgE-mediated allergies [13].

SPT has advantages such as low cost, minimal invasiveness, rapidity of performance and good diagnostic value when performed skillfully and in a standardized manner [1,14]. There is a number of somewhat aligned guidelines for performing and interpreting SPT [13,15–18]. The present study follow the guidelines of the European Academy of Allergy and Clinical Immunology (EAACI) [13,19]. A vital part of the SPT procedure is the use of both a positive and a negative control [8]. These are included in the assay: to prove that the skin is capable of producing a reaction [19], to assess whether reactivity to histamine is correlated with sensitivity to the tested allergens [20], to detect insufficient lancet penetration leading to false negatives, to brisk lancet handling resulting in false positives (false negative/positive IgE sensitization status), and to prevent e.g. patients suffering from dermatographic urticaria from being falsely diagnosed with an allergy [15,16,21]. In the positive control histamine dihydrochloride 10 mg/mL is usually used to directly induce wheal and flare responses principally bypassing the immunological system [13,22,23]. A negative control typically consists of the allergen solution vehicle, most commonly glycerinated saline is applied [16].

It is well documented that skin reactivity measured by histamine- and allergen-induced skin prick reaction varies considerably. This is likely in part related to subject-adhered factors such as age [24,25], gender [26,27], concomitant medication [13,28], and seasonal variability [26,29]. Besides the importance of the extract composition and potency, other SPT-influencing factors are related to the technique itself, i.e. different procedures (including the practice of adjustment to the histamine reactivity) [30–32], the chosen body region [26], the distance between each skin pricks and the SPT lancet/device [1,33–37]. Lastly, the lancet weight utilized during SPT is often cited as being an important parameter in the procedure [1,13,38], but little evidence support this notion [39] and no attempts have been made at quantifying an appropriate lancet weight range. *Østerballe et Weeke* (1979) and others [40,41] have examined the effect of the depth of the lancet pressed into the skin and found that the wheal size increased until the prick depth reached 1 mm contributing to the design of the 1 mm tip shouldered standard SPT lancet [40]. Following this, a number of studies have attempted to optimize the lancet design but not accounted for lancet weight applied to the skin during the SPT [14,35,41]. Since it is desirable to increase the diagnostic precision and reliability of the procedure, while decreasing discomfort and incidents of bleedings [8,35,42], the present study explored the relationship between the lancet weight applied during histamine SPT and wheal reactions in healthy volunteers with no history of allergy. Additionally, flare response intensity, prick-evoked pain, and frequency of bleeding were monitored.

## Methods

The study was designed to examine whether the lancet weight applied in SPT affects the wheal and flare response, pain, and incidents of bleeding. Based on initial pilot studies using a range of lancet weights, four different lancet weights where chosen; 25 g, 85 g, 135 g and 265 g. These were applied repeatedly on the forearms and on the back of the 20 participating subject. Two different histamine dihydrochloride concentrations were used; 1 mg/mL (equivalent to 0.1% or 5.43 mmol/L) and 10 mg/mL (equivalent to 1% or 5.43 mmol/L) as positive controls and a saline solution similar to that of the two histamine solutions was used as a negative control. All applied solutions (both histamine concentrations and the negative control solution) were 50% glycerinated (0.9 g/L saline and 1.0 g/mL glycerin).

### Subjects

In total 10 females and 10 males (age: 22.8 ± 2.2, range: 19–25 years) participated in one experimental session lasting approximately two hours. Healthy females and males in the age of 18–60 years were eligible for enrolment in the study. The exclusion criteria were: any present or previous dermatological diseases and any history of allergy, pregnancy, drug addiction defined as the use of cannabis, opioids or other drugs, previous neurologic, musculoskeletal or mental illnesses, and lack of ability to communicate and/or cooperate. All the subjects had to sign the declaration of consent before participating in the study. The study protocol was approved by the local ethics committee of Region Nordjylland, Denmark (approval number: N-20140078). Enrolment of subjects for study occurred between the 25^th^ of January 2015 and the 9^th^ of March 2015.

### Experimental setup

On the central volar aspects of the forearms eight squares with diagonals of 40 mm were marked with a metallic paint marker (054, Edding International GmbH, Germany) with a distance of 3 cm from the wrist and 3 cm from the antecubital fossae (see illustration of setup in S1 File). This setup is in line with the European recommendations of SPT standardization [13]. For an overview of the current European, American and Australian guidelines on the SPT procedure and interpretation please refer to S1 Table. On the back 12 squares with diagonals of 40 mm were marked with a metallic marker. Four squares were placed in a longitudinal line 20 mm lateral to the vertebra on both sides. Lateral to the two lower squares, two identical squares were marked. Droplets of histamine and saline were placed in the centre of each square. The concentrations of histamine were 1 mg/mL and 10 mg/mL (EAACI recommended positive control [19]) and saline was used as a negative control (default preparations, Allergopharma, Diagenics, Bletchley, UK). On the forearms of each subject a total of four histamine pricks and four saline pricks were conducted and assessed whereas on the back a total of eight histamine pricks and four saline pricks were conducted and assessed. In the entire experiment 400 weight-controlled skin pricks were conducted (for each subject: eight on the forearms, 12 on the back). All skin pricks were conducted according to right-left mirrored proximal-distal allocation schedules in which both anatomical location, order of applied weight and choice of solution was included. The study was single-blinded so that the subjects were unaware whether histamine 10 mg/mL, 1 mg/mL or saline were being applied. Please refer to S1 Table for further on the setup and allocations schedules.

### Performing the SPT

**Fig 1:**
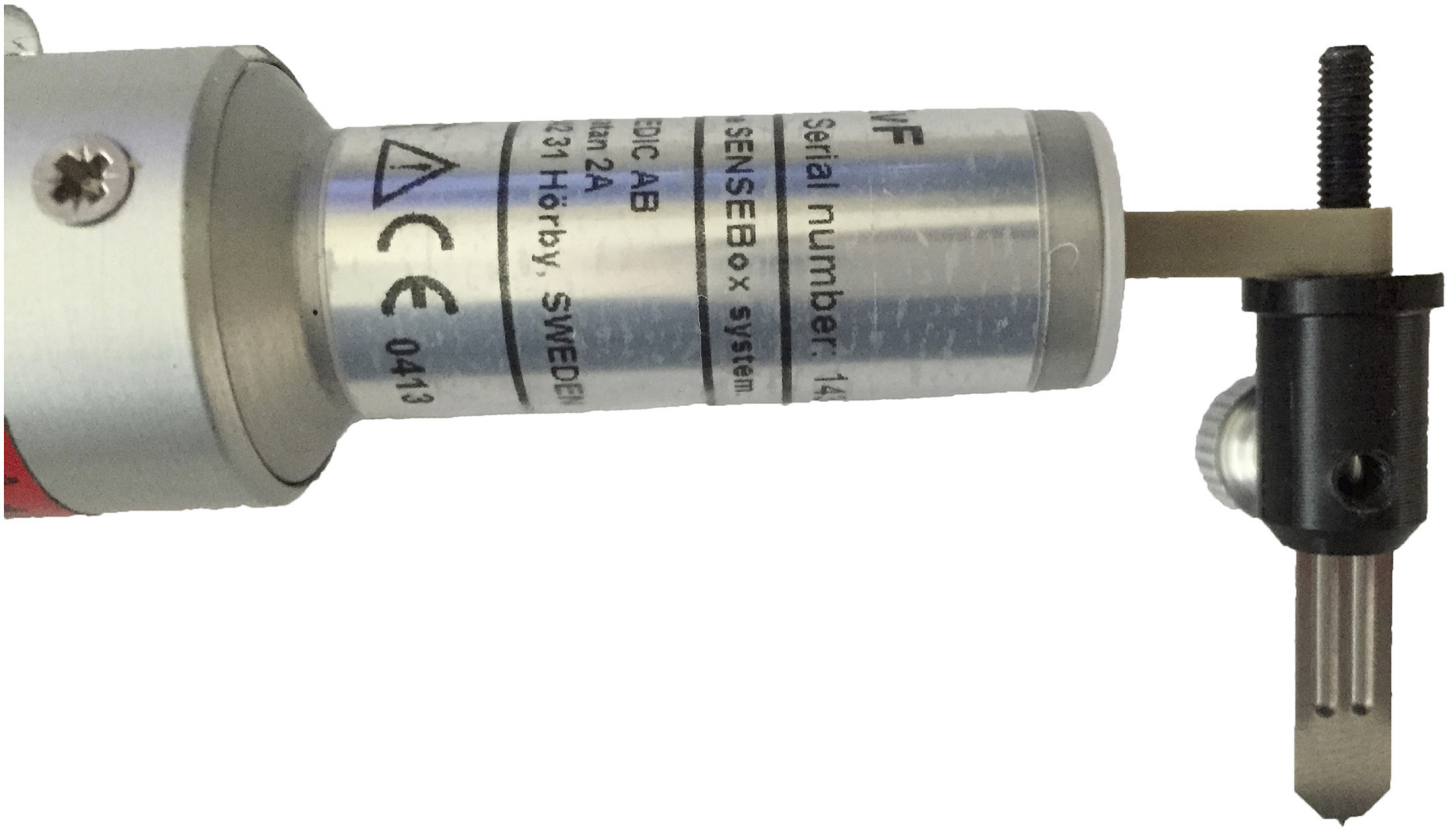
The setup of the SPT lancet attached to the SENSEBox Electronic von Frey weight transducer.

The pricks were performed with four different lancet weights; 25 g, 85 g, 135 g and 265 g. To control the different lancet weights a SENSEBox transducer (Somedic, Hörby, Sweden) was used. The setup provided a computerized weight profile output sampled at 100 Hz and was manually calibrated before use. A sterile standard lancet with a 1 mm sharp tip and with horizontal shoulders (Allergopharma, Diagenics, Bletchley, UK) was attached to the weight transducer using a custom made SPT lancet mount (Aalborg University, Aalborg, Denmark), see Fig 1, below.

The lancet was pressed through a droplet of histamine or saline perpendicularly to the predetermined area of skin. This weight-monitoring approach resulted in a very consistent lancet weight performance for each application lasting approximately 1.5–2 seconds, with a predetermined peak intensity being applied for approximately 1 second. The exact peak lancet weight was subsequently extracted from the weight profile output.

### Physiological data

Ten minutes after the first prick was performed images were captured with a Dino-Lite Pro digital dermascope polarizer with a resolution of 1.3 megapixel (Dino-Lite, Naarden, Holland) to identify incidents of bleeding not readily visible by regular visual inspection. The speckle contrast flowmetry (MoorFLPI, Moor Instruments, Devon, UK) were performed to measure the mean superficial perfusion. The distance between the speckle contrast flowmetry and the application area was 45 cm, the total exposure time was 8.3 ms and the gain setting was 150 units. The images from the speckle contrast flowmetry were analysed on appertaining software (MoorFLPI Review V 4.0, Moor instruments) to assess the flare intensity within the predefined squares. Using an averaged region of interest approach the mean superficial perfusion for each square was found. Additionally, the diameters of the wheals were measured on the longitudinal diameter and on the orthogonal midpoint diameter with a transparent ruler with mm resolution. One investigator measured the wheal diameters in the same order that the SPTs had been applied and the lack of wheals, e.g. in response to saline was noted.

### Psychophysical data

For each prick the evoked pain intensity rating was monitored using an electronic visual analog scale (VAS); SENSEBox VAS (Somedic, Hörby, Sweden). The VAS-ratings were sampled at 100 Hz and the subjects were notified immediately before each skin prick. The modified VAS-scale ranged from 0–100 mm where 0 mm indicated "*no pain*", 50 mm indicated "*worst imaginable pain caused by a needle*" and 100 mm indicated "*worst imaginable pain*". The individual peak pain VAS-scores for each prick was extracted from the stimulus response curves.

### Sub-experiment of SPT weight recordings in clinicians

SPT weight was sampled from four clinicians routinely working with SPT at the Allergy Clinic, Gentofte Hospital, Gentofte, Copenhagen, Denmark. For lancet weight measurements the setup shown on Fig 1 was reversed so that a 1.5 x 1.5 cm square of artificial suturing practice skin (Aphelion Supplies Ltd., Birmingham, UK) was attached to the SENSEBox Electronic von Frey weight transducer. The four clinicians were instructed to conduct a series of six skin pricks, with standard 1mm lancets, resembling, as closely as possible, those applied on patients in regular clinical routine. SPT weight profiles were sampled at 100 Hz and subsequently analysed for the peak weight value. It should be highlighted that these data are from a very limited and potentially non-representative sample and merely attempts to preliminarily probe issues pertaining to SPT lancet weight in the clinical routine.

### Statistical analysis

Data handling and calculation of descriptive statistical parameters were conducted in Excel (Microsoft, Redmond, Washington, USA). Further, statistical analyses were performed in SPSS (IBM, Armonk, New York, USA). The graphical representations were created with GraphPad Prism 6 (Graphpad Software, Inc, La Jolla, California, USA). Sample size calculations were conducted based on previously obtained test-retest reliability data of responses to histamine applied using SPT lancets [43] and using the approached outlined in [44] for crossover designs. A α-level of 0.05, a power of 80%, and a least relevant difference of 20% were applied giving rise to a minimum required sample size estimate of ≈18. To account for potential dropout or no-show a total of 20 subjects were recruited. Data is generally presented as arithmetic means ± standard error of the mean (SEM), unless else is specifically stated. The obtained data for wheal diameter were confirmed to be normally distributed within the various experimental conditions by visual inspection of Q-Q plots and frequency distribution. Additionally, all the acquired metric data variables, apart from peak pain scores, were normally distributed, according to the Shapiro-Wilk test. The obtained data for wheal diameter were tested by One-way Repeated Measures ANOVA and a Tukey post-hoc test was used to observe inter-group differences and to correct for multiple comparisons. The obtained data for the superficial perfusion were tested by using a paired sample *t*-test to observe the difference between the data from the baseline (i.e. superficial blood-perfusion monitored by FLPI provides a baseline value) and the obtained data after performing the SPT. To observe the difference between groups and correct for multiple comparisons a Tukey post-hoc test was used. The collected data for the mean peak pain VAS-score were tested by a Wilcoxon rank-sum test. For the qualitative data, the Chi-square (Χ^2^) test was used to determine whether the number of pricks that caused bleeding incidents were significantly associated with the applied lancet weight. For the data obtained in the sub-experiment of SPT weight recordings in clinicians and for the applied lancet weights only descriptive statistics were performed to elucidate data variability, i.e. the mean and the coefficient of variation (CV = standard deviation expressed as % of the mean). A *p*-value < 0.05 was considered significant and marked with a single asterisk (*) in legends, while a *p*-value < 0.01 is marked with double asterisk (**).

## Results

All enrolled subjects (n = 20) completed the experiment with no serious and/or unexpected adverse events. Only on two separate occasions did SPT with saline and a lancet weight of 265 g cause wheal reactions (<3 mm diameter). Subject no. 3 had a mean wheal diameter of 2 mm on the forearm while subject no. 19 had a mean wheal diameter of 2 mm on the back after SPT with saline. No wheal reactions occurred in response to saline in weights < 265 g. In this study, no significant differences were observed in relation to gender or anatomical location of any of the monitored reactions, however, since this was not the study objective, statistical powering were likely inadequate for this purpose.

### Applied SPT lancet weights

Applied SPT weights generally had very low variability. The mean and the CV for the peak of the four pre-established lancet weights; 25 g, 85 g, 135 g and 265 g, are presented in Table 1. Notice that a tendency towards higher applied weights on the back was observed particularly for the 265 g condition.

**Table 1:** The mean and coefficient of variation (CV %), calculated as the ratio between the standard deviation and the mean for the four predetermined peak lancet weights; 25 g, 85 g, 135 g and 265 g at the forearm and back. Data for applied lancet weight are pooled for conditions with saline, 1 mg/mL of histamine and 10 mg/mL of histamine. Notice that the deviation is proportionally higher in the 25 g weight group.

Location	Weights			
	25 g	85 g	135 g	265 g
**Forearm**	26.3 (4.6%)	86.6 (1.7%)	133.2 (1.4%)	260.4 (1.5%)
**Back**	27.63 (2.2%)	89.9 (1.1%)	133.6 (1.2%)	270.0 (1%)

### Higher lancet weight causes larger wheal diameter

**Fig 2:**
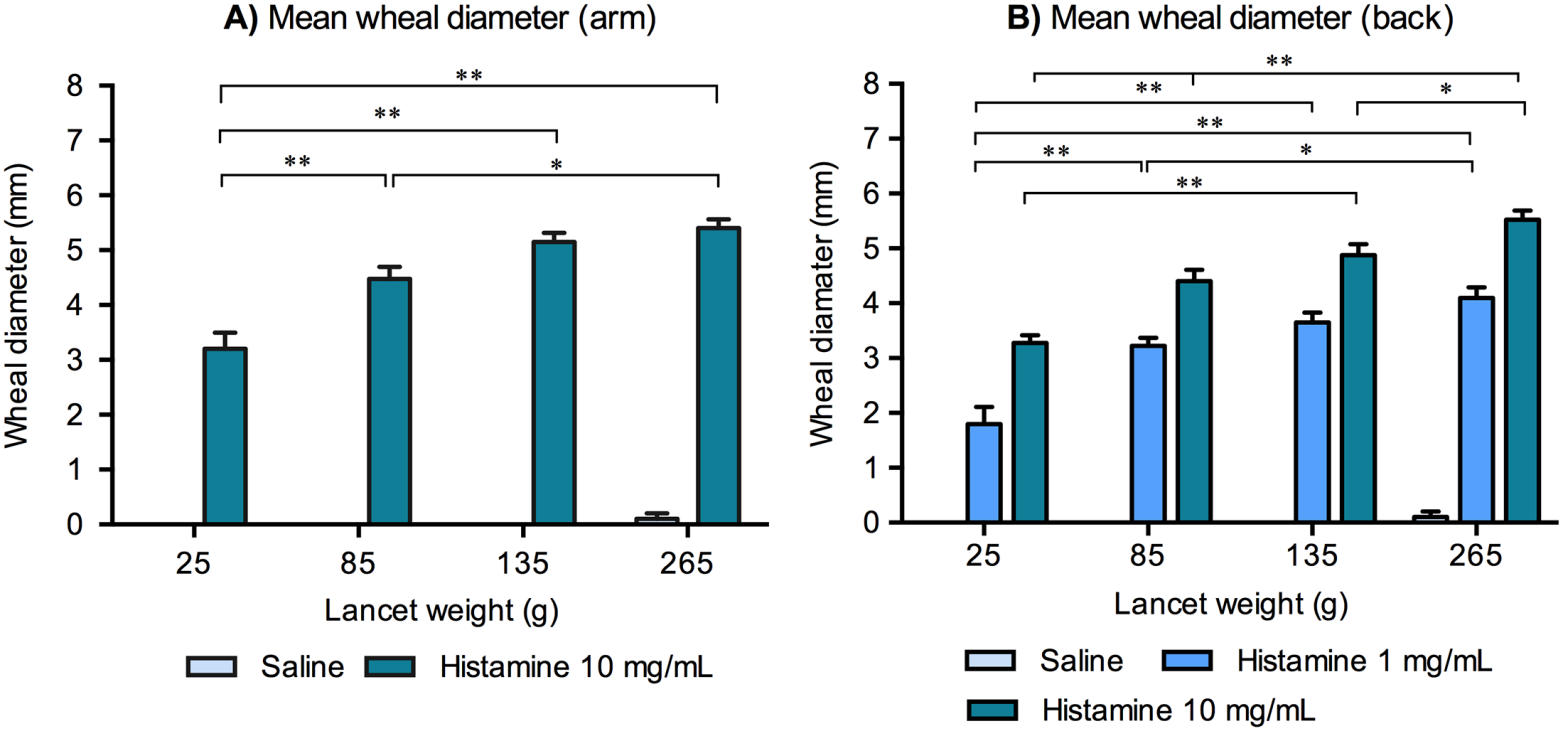
**A)** The mean wheal diameters on the forearms for each lancet weight; 25 g, 85 g, 135 g and 265 g. A higher lancet weight significantly increased the sizes of the wheal reactions. **B)** The mean wheal diameters on the back for each lancet weight; 25 g, 85 g, 135 g and 265 g. A higher lancet weight significantly increased the sizes of the wheal reactions. A significant difference was also observed when comparing the two histamine concentrations: 1 mg/mL and 10 mg/mL. For each condition; n = 20. * = p < 0.05, ** = p < 0.01. Mean ± SEM.

The mean wheal diameter increased significantly with higher lancet weights when applying histamine regardless of concentration and anatomical location (see Fig 2A and 2B). Using a histamine concentration of 10 mg/mL on the volar aspects of the forearms, a difference was seen in the mean wheal diameters between 25 g and the three other applied lancet weights; 85 g, 135 g and 265 g (*p* < 0.01). Furthermore, a difference was observed in the mean wheal diameters between the lancet weights of 85 g and 265 g, see Fig 2A. Oppositely, when applying saline no significant difference in the mean wheal diameters was seen between the four lancet weights; 25 g, 85 g, 135 g and 265 g on the forearms nor on the back.

At a lancet weight of 25 g and a histamine concentration of 10 mg/mL the mean wheal diameter on the volar forearm was 3.2 mm, however 4/20 subjects had a wheal diameter < 3 mm, thus not constituting positive reactions by most current guidelines [13,19]. The 99% CI for the mean wheal diameter at a lancet weight of 25 g was 2.4–4.0 mm. However, the lancet weights of 85 g, 135 g and 265 g resulted in lower boundaries of the 99% CI’s consistently > 3 mm. When applying a histamine concentration 1 mg/mL there was a difference in the mean wheal diameter on the back between the lancet weight of 25 g and the other three used lancet weights; 85 g, 135 g and 265 g (*p* < 0.01). Moreover, a difference was observed for the mean wheal diameters between the lancet weights of 85 g and 265 g, see Fig 2B.

In addition, at a histamine concentration of 10 mg/mL a difference was found in the mean wheal diameter between the lancet weight of 25 g and the other three used lancet weights 85 g, 135 g and 265 g (*p* < 0.01). Furthermore, a difference was seen between 85 g and 265 g and between 135 g and 265 g, see Fig 2B. SPT on the back when applying histamine 10 mg/mL at a lancet weight of 25 g resulted in a mean wheal diameter of 3.3 mm. However, 2/20 subjects had a wheal diameter < 3 mm and the 99% CI for the mean wheal diameter was 2.9–3.9 mm. With lancet weights ≥ 85 g the lower boundary of the wheal diameter 99% CI was always > 3 mm in the 10 mg/mL group. A lancet weight of 135 g was required to consistently obtain a lower 99% CI boundary > 3 mm when histamine 1 mg/mL was applied in the SPT on the back.

### Higher lancet weight causes increased flare reactions

**Fig 3:**
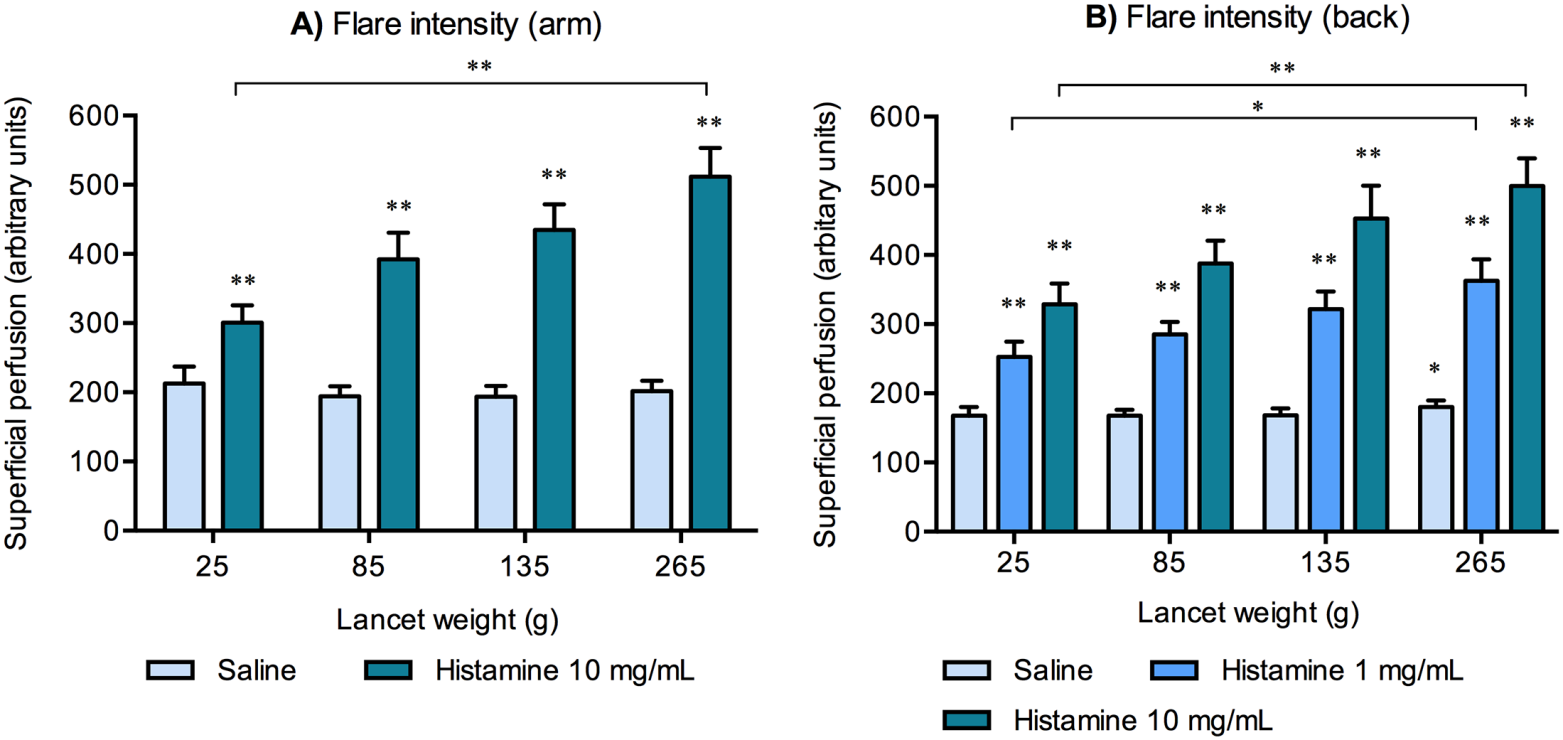
**A)** Changes in the mean superficial perfusion on the forearms according to the lancet weights: 25 g, 85 g, 135 g and 265 g. A difference in the mean superficial perfusion was seen from baseline to all the four applied lancet weights. **B)** On the back a difference in the mean superficial perfusion was seen from baseline to all the four applied lancet weights with both histamine concentrations (1 mg/mL and 10 mg/mL) and from baseline for the lancet weight of 265 g with saline as well. For each condition; n = 20. * = p < 0.05, ** = p < 0.01. Asterisks (*) or (**) placed just above the bars indicates significant difference from baseline superficial skin perfusion. Mean ± SEM.

An increase in the mean superficial perfusion in accordance with increased weight when applying histamine 10 mg/mL was seen only from the lancet weight of 25 g to 265 g on the forearms (*p* < 0.01) yet from the baseline a difference in the mean superficial perfusion was seen for all used lancet weights; 25 g, 85 g, 135 g and 265 g, see Fig 3A.

A significant increase was observed in the mean superficial perfusion between the lancet weights of 25 g and 265 g when applying 1 mg/mL versus 10 mg/mL histamine on the back. Furthermore, a difference from baseline was found for 25 g, 85 g, 135 g and 265 g (*p* < 0.01) both when applying histamine 1 mg/mL and histamine 10 mg/mL. Notice when applying saline a difference was also seen from baseline levels to 265 g skin pricks (see Fig 3B).

### Higher lancet weight causes increased occurrence of bleeding and increased provoked pain perception

**Fig 4:**
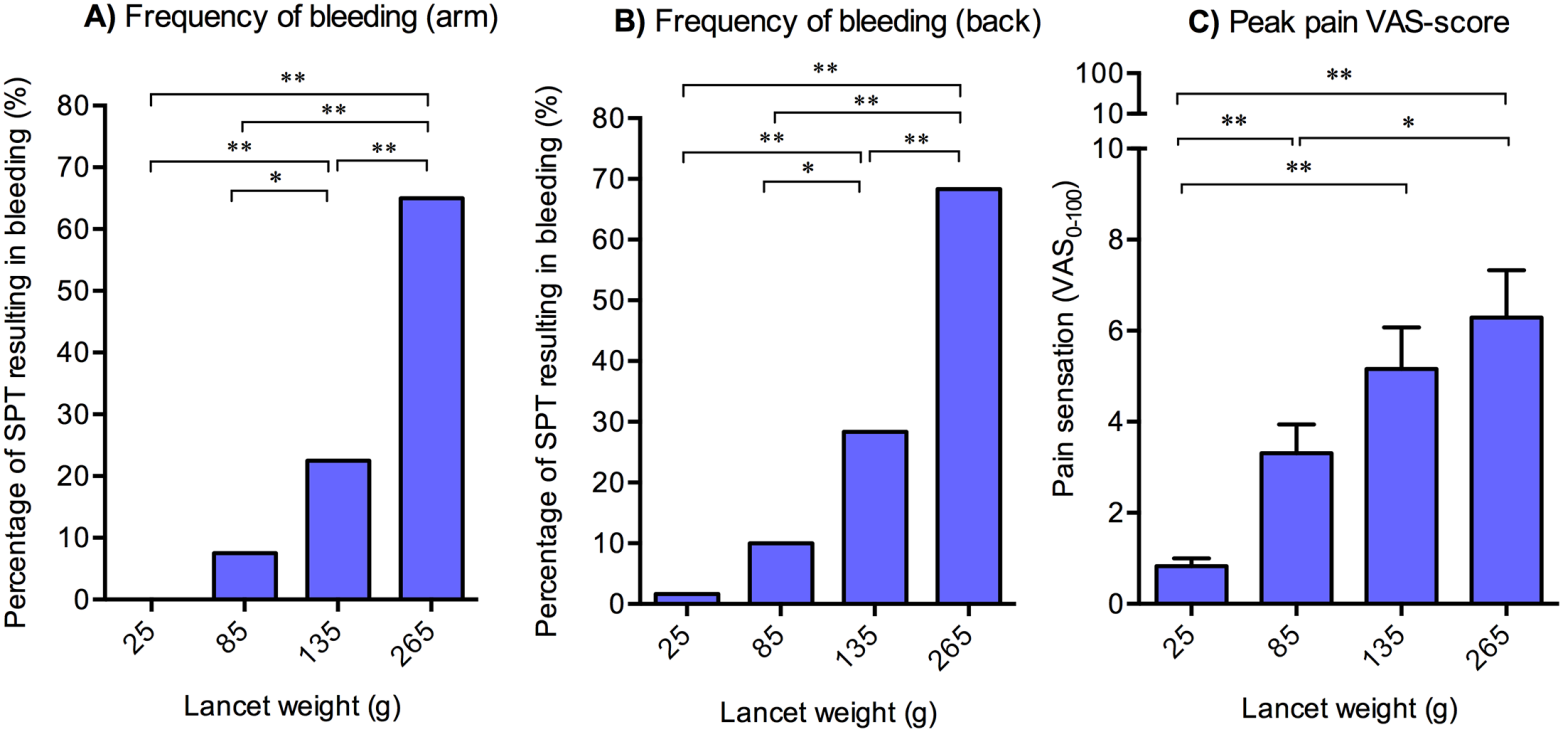
**The percentage distribution of bleeding incidents in response to individual SPTs when using the four lancet weights; 25 g, 85 g, 135 g and 265 g** at **A)** the arm and **B)** the back, regardless of the applied solution (saline, 1 mg/mL histamine or 10 mg/mL histamine). A higher lancet weight caused increased incidents of bleeding. **C)** Peak pain intensity in response to the four lancet weights for both arm and back rated on a coVAS. The peak pain intensity increased significantly with increased weight. Notice that pain during SPT, regardless of weight, remains negligible, i.e. < 8 on a 0–100 VAS (N.B.: discrete x-axis is applied for graph C). For each condition; n = 20. * = p < 0.05, ** = p < 0.01. C) Mean ± SEM.

A higher lancet weight was found to cause more incidents of bleeding both on the forearms and on the back (*p* < 0.01), see Fig 4A and 4B. Significant differences in incidents of bleeding were found between 25 g and 135 g, 25 g and 265 g, 85 g and 135 g, 85 g and 265 g, and 135 g and 265 g both on the forearms and on the back (see Fig 4A and 4B). Furthermore, no differences were observed when comparing the number of incidents of bleeding on the forearms with the number of incidents of bleeding on the back.

Expectedly, higher lancet weights were associated with increased, albeit still only mild, pain. A difference in the mean peak VAS-score for pain was seen between 25 g and the three other applied lancet weights; 85 g, 135 g and 265 g. Moreover, a difference between 85 g and 265 g was found, see Fig 4C. No significant difference was observed in the peak pain between the arms and the back.

### Preliminary data suggest clinical staff exert SPT lancet weights characterized by high inter-variability

**Fig 5:**
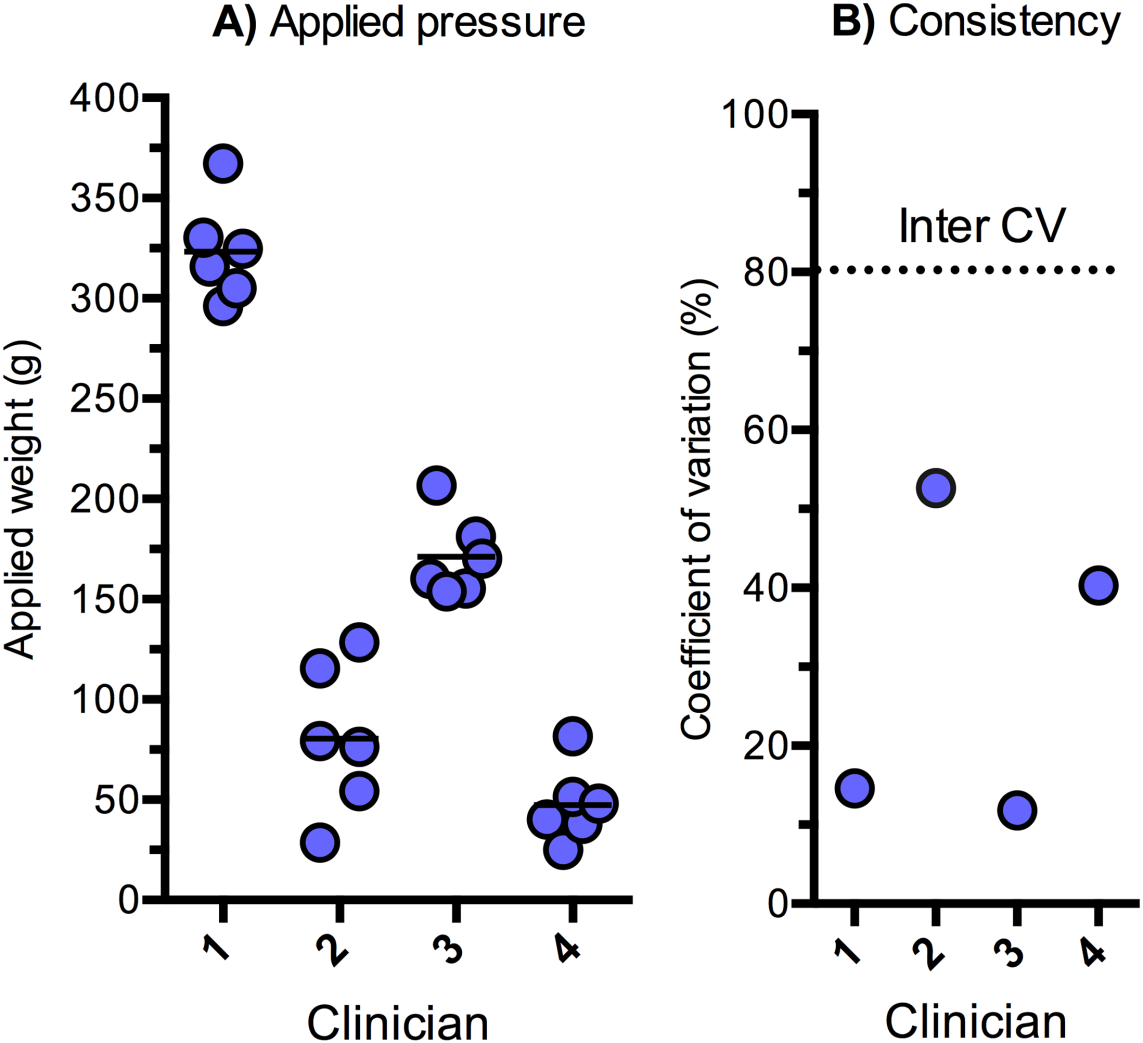
**A)** Shows individual lancet peak weight measurements from four different clinicians (no. 1–4). The horizontal lines denote the arithmetic mean for each individual. **B)** Shows intra-reliability and inter-reliability (dotted horizontal line) of applied weight expressed as the coefficient of variation (standard deviation expressed as % of the mean) for each of the four testees and between the four testees. CV% = Coefficient of variation (calculated as the ratio between the standard deviation and the mean in %).

From sampling in a small cohort of clinicians working with SPT the preliminary data suggests that inter-reliability is poor, varying between 29 g and 367 g of weight (mean CV = 81%), see Fig 5A. However, intra-reliability is generally better, particularly for clinician no. 1 and no. 3, see Fig 5B.

## Discussion

The present study shows that higher SPT lancet weights increase the mean diameter of wheals, intensify the flare, increase the peak lancet provoked pain, and causes more frequent bleedings. Additionally it was elucidated that lancet weights of ≈ 25 g is generally insufficient to reliably induce adequate positive wheal responses (i.e. > 3 mm in diameter according to most current guidelines [13]) while 85 g of lancet weight was found to be sufficient. Ideally the SPT procedure should be without false negative and false positive results, give rise to no adverse events, entail limited patient discomfort and generally not result in bleeding [13,19,27,35,38,42]. To pursue the ideal SPT protocol the variables related to the procedure should be adequately optimized and controlled, e.g. the prick device geometry, the concentration of allergens (and histamine) and the applied lancet weight are three frequently mentioned parameters [35,39]. Of the three parameters both composition of allergens and lancet design have received significant attention [1,33,35,40,45], while this study is the first to quantify lancet weight in relation to skin reactivity. The current study indicates an usefulness of locking lancet weight in a predetermined ideal range (presumably between >25 g and <85 g using 10 mg/mL histamine) or adjusting lancet weight when training to perform SPT in the clinic. By doing so, it should be possible to reduce false positive and false negative results and additionally limit pain and incidents of bleeding. However, further research is warranted and this study is not exempt from limitations. Particularly, the present study did not apply allergens but histamine (commonly used as a positive control). This sidesteps the mast-cell degranulation process and it is unknown whether SPT reactions to actual allergens could potentially be less susceptible to aggravation by increased lancet weight. Lastly, it should be noted, that for the preliminary data subset recorded amongst the four clinicians, it has not been assessed to which extend the skin prick weight delivered to the Somedic weight transducer correlates with the skin prick weight delivered on human skin. On the other hand this study was conducted in a homogenous group of young healthy controls, wherein SPT responses were unambiguously intensified by higher lancet weight. In clinical practice a number of uncontrollable variables are known to affect SPT including, gender, age, ethnicity and season [20,26,27], thus making it highly desirable to adjust for as many influencing controllable factors as possible.

### Higher lancet weight is associated with larger wheals, increased frequency of bleeding and more pain

A few previous studies have probed the frequently held notion of lancet weight as being important for the SPT procedure, but no studies until now have quantified various SPT lancet weights and their resultant skin responses. A study by *Phagoo et al*. (1991) actually elucidated that weight could be a vital parameter in SPT by using undefined: “*light*”, “*moderate*”, and “*hard*” application weights and subsequently monitoring wheal reactions. However, the study failed to quantify both the used weight ranges and the individual weight application groups, making the results very limited in terms of extrapolatability [39]. *Østerballe et Weeke* (1979) showed that an increased prick depth correlated with an increased wheal diameter [40] contributing to the development of the 1 mm shouldered SPT lancet. Since histamine acts on the local vascular layer [46] it should be exposed localized to the micro-vascular system with the upper plexus approximately 1–1.5 mm below the upper layer of epidermis [47,48]. The present study showed a significant stepwise increase in the mean wheal diameter in response to higher lancet weights when applying histamine. That is, despite using a standardized shouldered, 1 mm tip lancet design, higher lancet weights (≥ 265 g) are still likely to drastically increase tissue exposure [39] resulting in larger skin reactions, bleedings [40] and an increased risk of false positives [8,13,35] while very low lancet weight, i.e. ≤ 25 g inadequately exposes tissue to histamine (and likely allergens) resulting in insufficient SPT reactions or in clinical practice; false negatives.

Furthermore, when performing SPT a secondary goal is to reduce patient discomfort such as pain [1,35]. As inherently expected [49,50], higher lancet weight significantly increased the pain experienced during the SPT procedure. By predetermination of an optimal lancet weight, which allows for high diagnostic sensitivity and specificity, unnecessarily high lancet weights (see Fig 5A and 5B) can be avoided, thus reducing the associated pain. This study warrants further research of the optimal lancet weight where the incidents of bleeding are as low as possible while still resulting in a positive wheal diameter > 3 mm. Based on the present results, a lancet weight in the range of > 25 g and ≤ 85 g seems to satisfy most practical clinical criteria for an ideal SPT procedure.

### Perspectives

**Fig 6:**
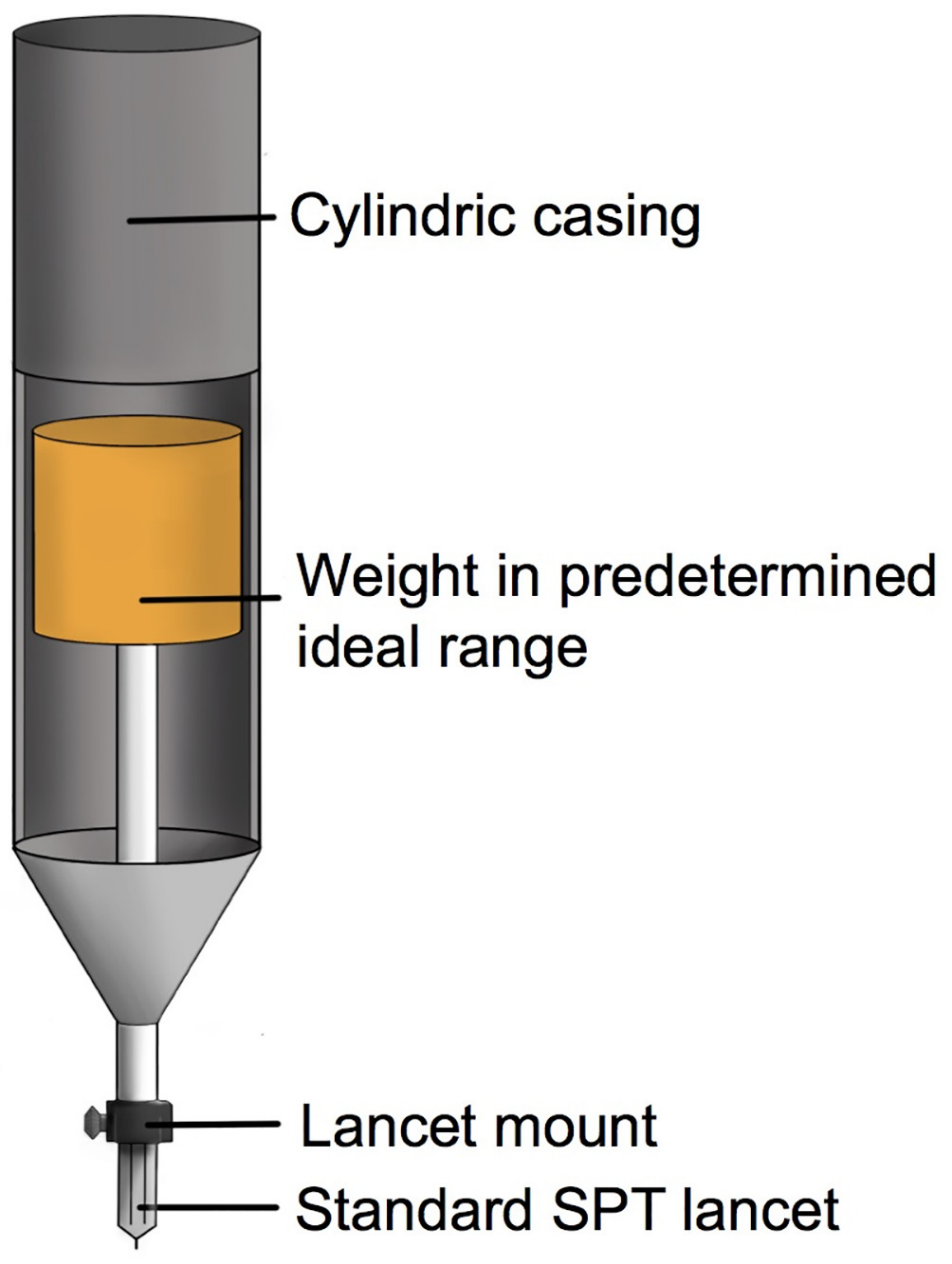
Weighted SPT lancet device. Depicts a suggested mechanical approach to a locked weight device allowing for SPT within a specific predetermined weight.

Higher lancet weights are positively associated with several unwanted aspects of SPT such as patient discomfort, risk of adverse events (although rare in response to SPT they do occur [51,52]), blood drawing and likely the incidence of false positives. Based on the findings of this study it is inherent to infer that a standardized SPT lancet weight could be within an optimum range balancing between false negative reactions and unwanted effects. This challenge could be overcome in the clinical setting either by supplying clinicians performing SPT with small gauge allowing them to periodically calibrate their exerted lancet weight. Another option is to use a small pinprick stimulator with a mount for detaching/attaching SPT lancet at a specific predetermined weight, e.g. as depicted on Fig 6. Further testing is needed to assure the feasibility of both approaches stated above.

The fact that increased lancet weights precipitates aggravated physiological responses, most prominently increases wheal and flare seems inherently logical and is likely a consequence simply of increased tissue exposure to, in this case, histamine. Despite it being a variable previously mentioned in the literature [1,13,38] this study is the first to quantitatively elucidate lancet weight as a potentially biasing variable and to document to which extent it influences SPT responses. In conclusion, with the presented limitations, this study provides novel and unambiguous evidence that higher lancet weights are associated with larger mean wheal diameters. This pose an important question for further investigation: can the lancet weight turn a true negative SPT to an allergen into a false positive or a true positive into a false negative and thus directly influence diagnosis?

## Supporting Information

S1 File Allocation schedules and anatomical setup for skin prick testing. Images 1–20 depict allocation of the areas and substances of skin prick testing on the volar aspect of the forearm and on the back.
(DOCX)

S1 Table An overview of international skin prick testing guidelines. An outline of various skin prick test guidelines.
(DOCX)
