# Supplementary material for: The Lancet Weight Determines Wheal Diameter in Response to Skin Prick Testing with Histamine
Source: PLoS One. 2016 May 23;11(5):e0156211. doi: 10.1371/journal.pone.0156211 (PMC4877047; doi:10.1371/journal.pone.0156211)
Supplement: S1 File — Images 1–20 depict allocation of the areas and substances of skin prick testing on the volar aspect of the forearm and on the back. (DOCX) [file pone.0156211.s001.docx]

***Supporting Information S1* - Randomization and anatomical setup for skin prick testing.**

### Setup on the forearms

| Setup 1 on the forearms | Setup 2 on the forearms |
| --- | --- |
| 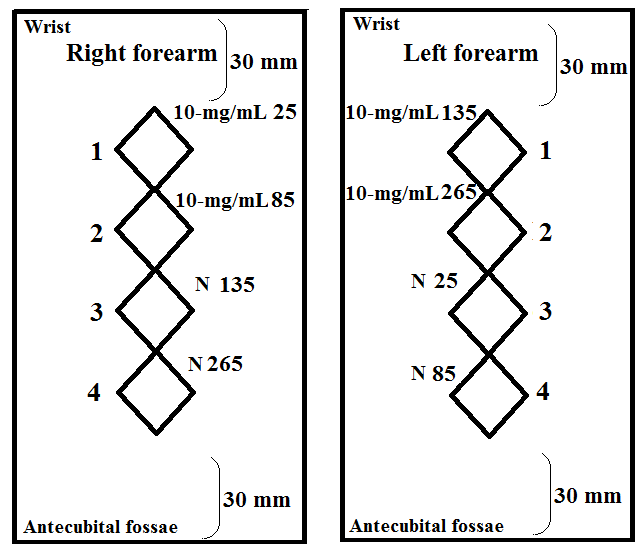 | 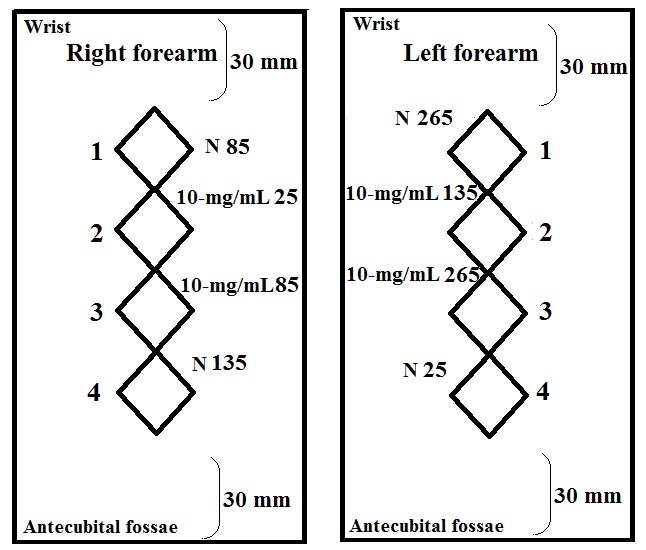 |
| Setup 3 on the forearms | Setup 4 on the forearms |
| 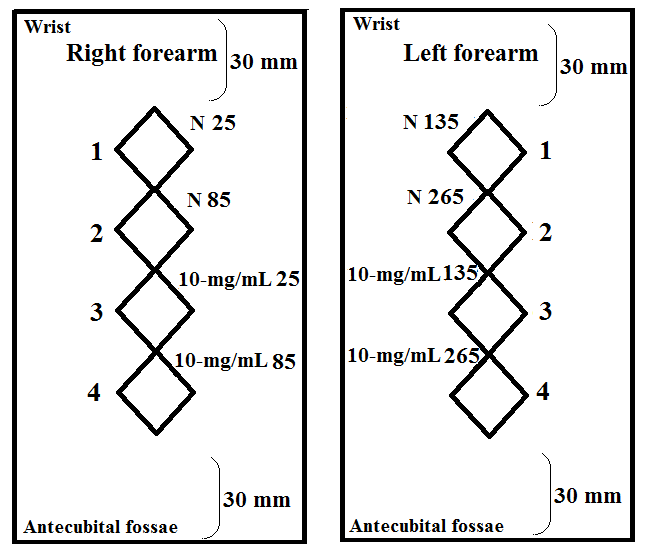 | 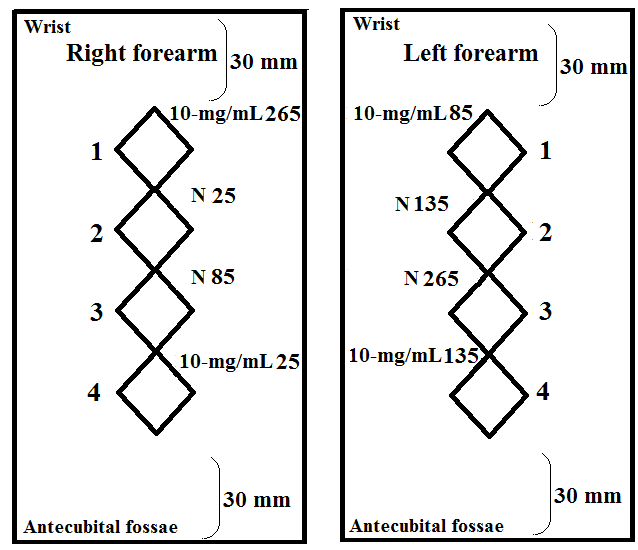 |

| Setup 5 on the forearms | Setup 6 on the forearms |
| --- | --- |
| 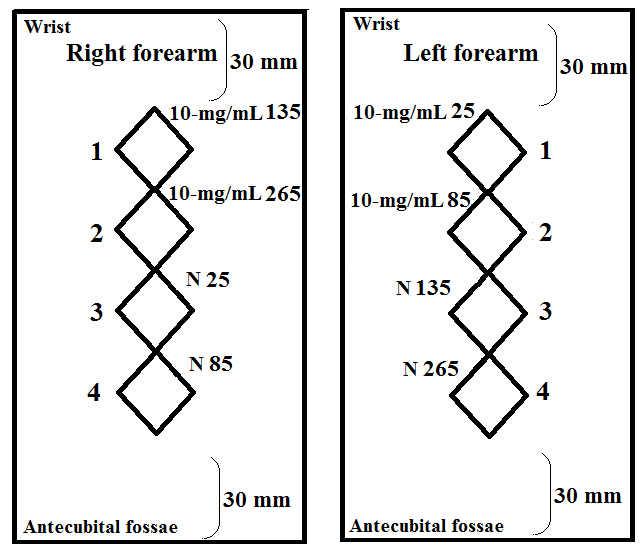 | 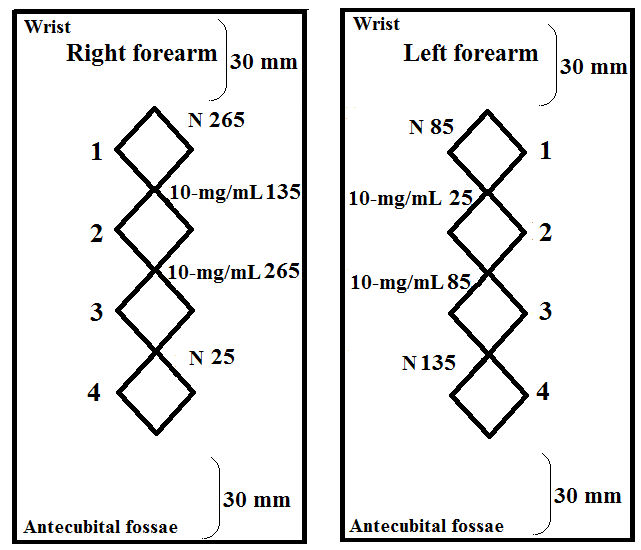 |
| Setup 7 on the forearms | Setup 8 on the forearms |
| 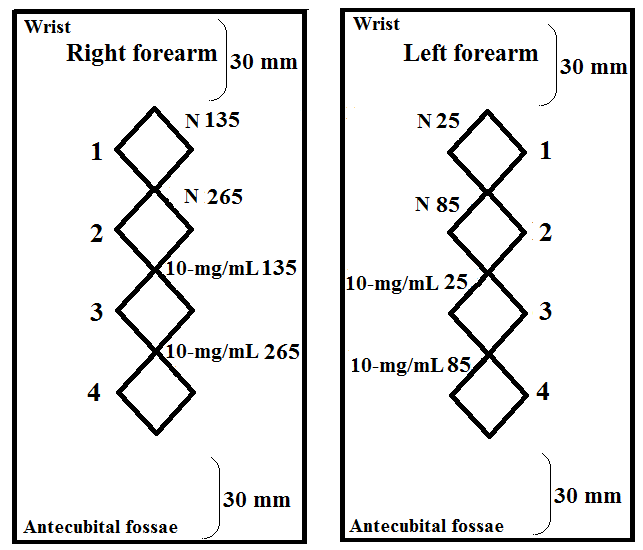 | 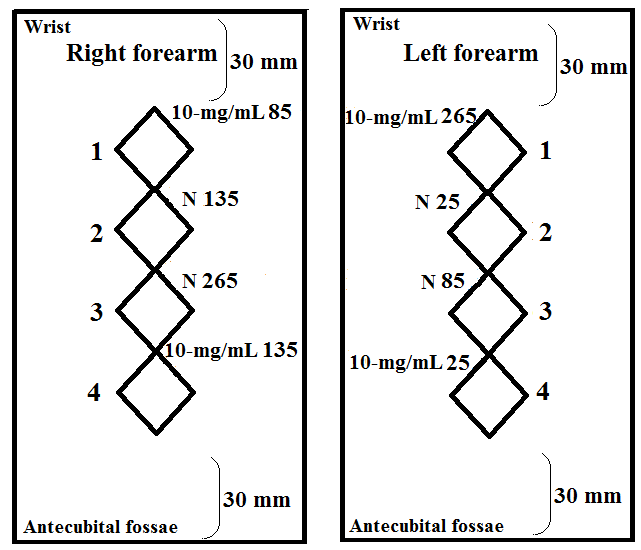 |

### Setup on the back

| Setup 1 on the back | Setup 2 on the back |
| --- | --- |
| 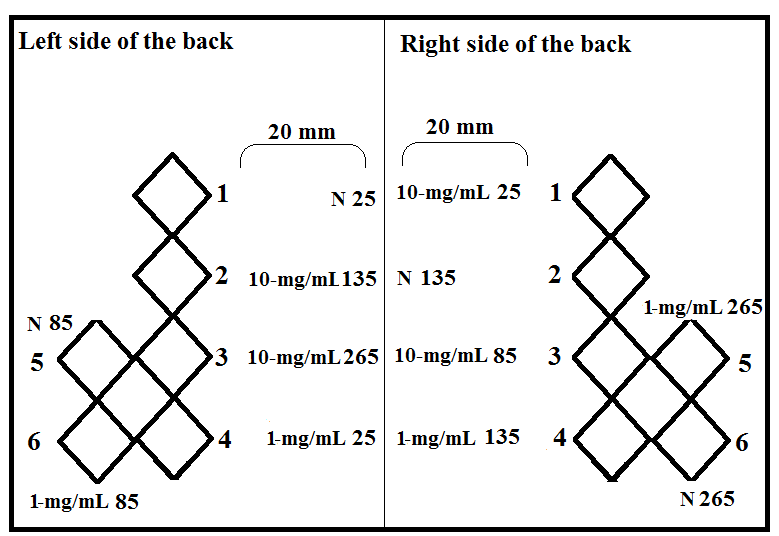 | 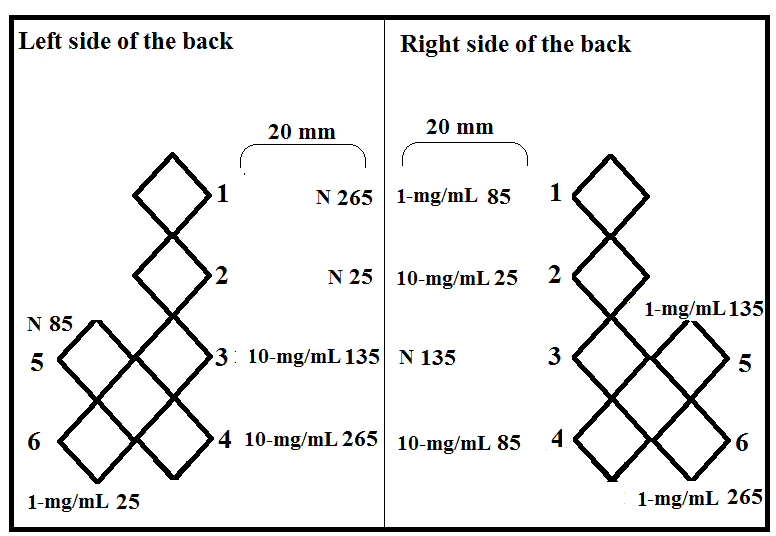 |
| Setup 3 on the back | Setup 4 on the back |
| 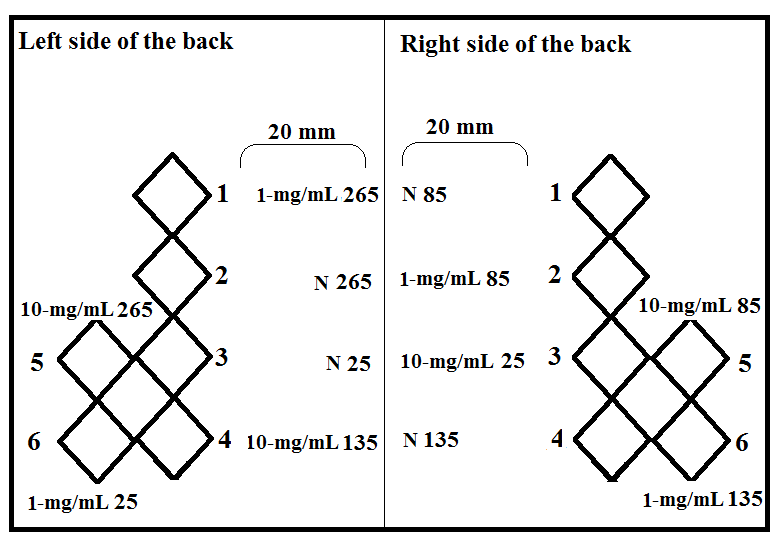 | 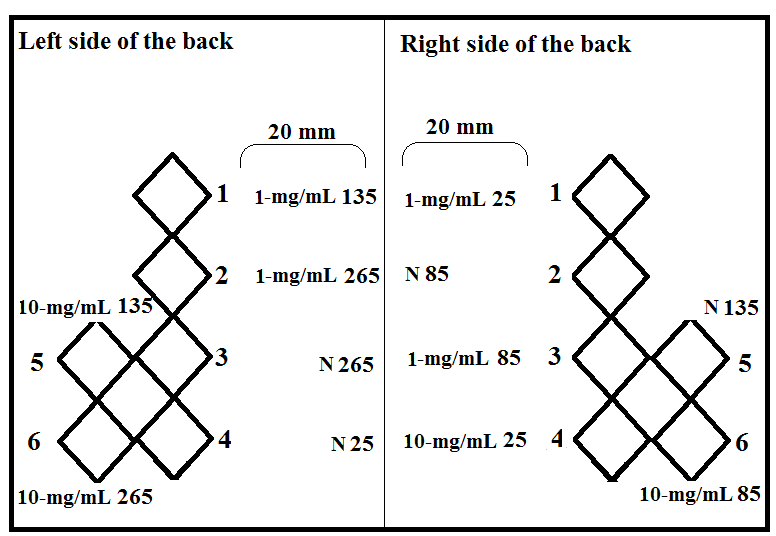 |
| Setup 5 on the back | Setup 6 on the back |
| 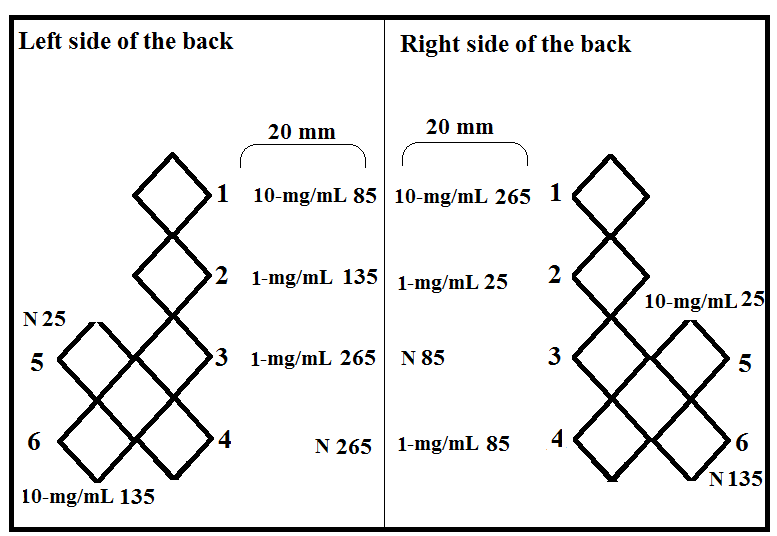 | 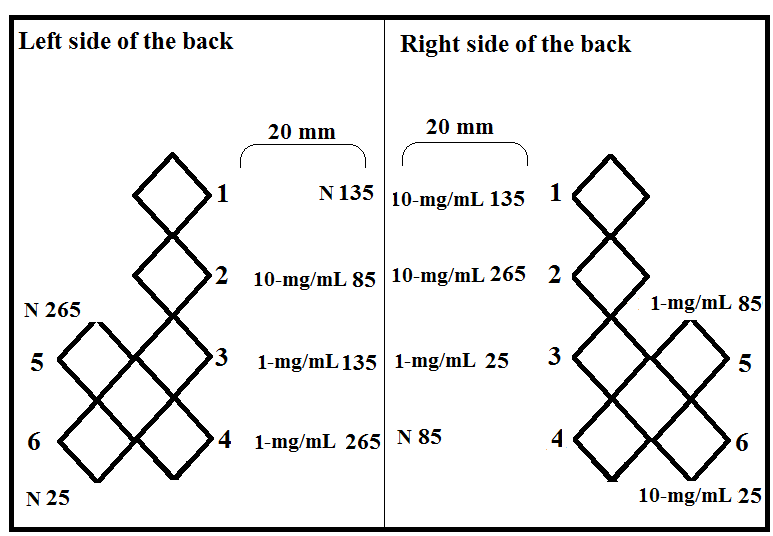 |

| Setup 7 on the back | Setup 8 on the back |
| --- | --- |
| 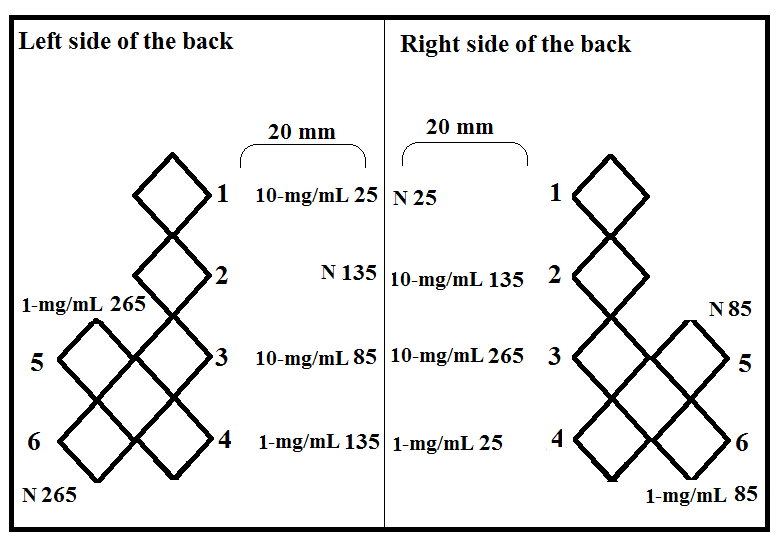 | 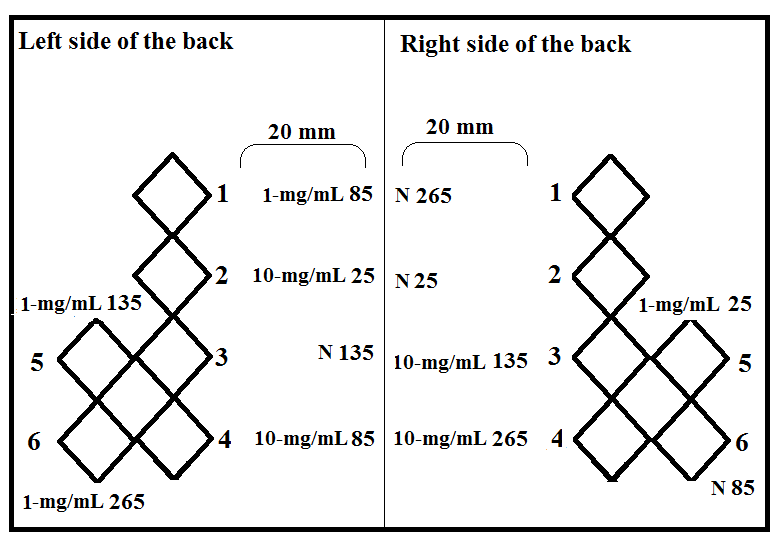 |
| Setup 9 on the back | Setup 10 on the back |
| 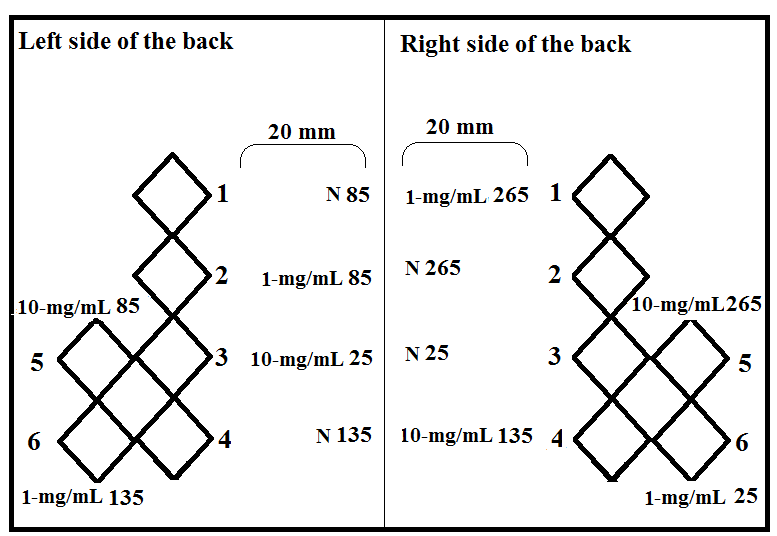 | 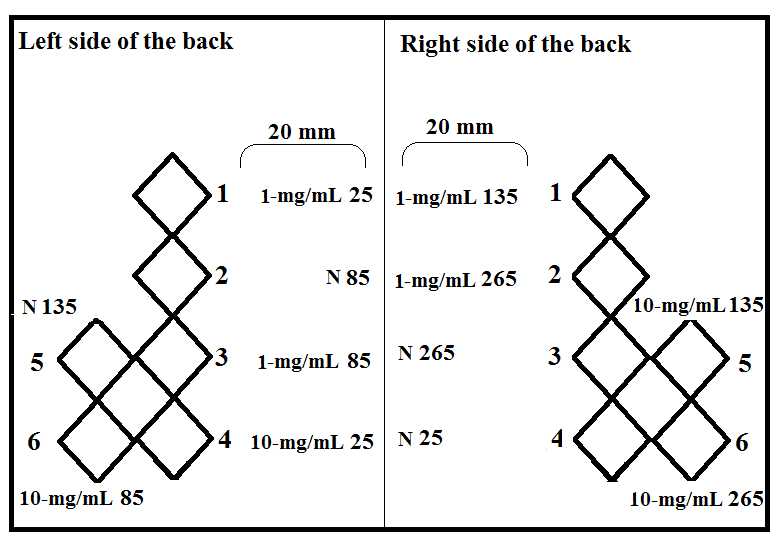 |
| Setup 11 on the back | Setup 12 on the back |
| 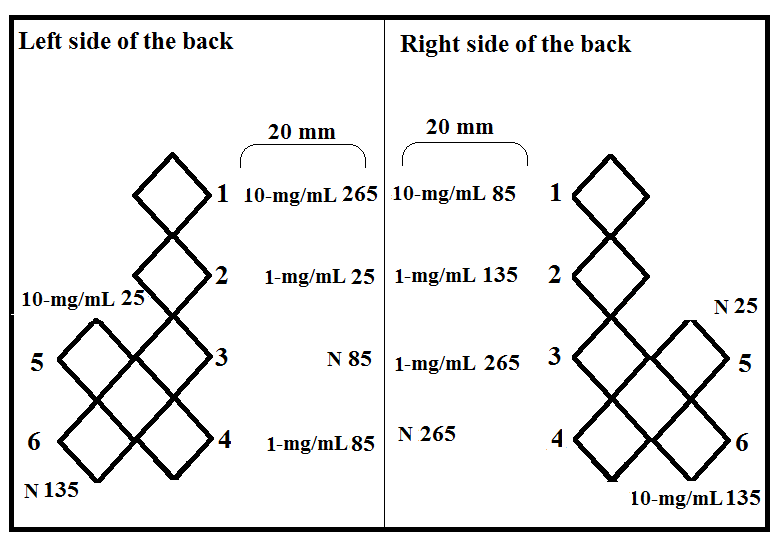 | 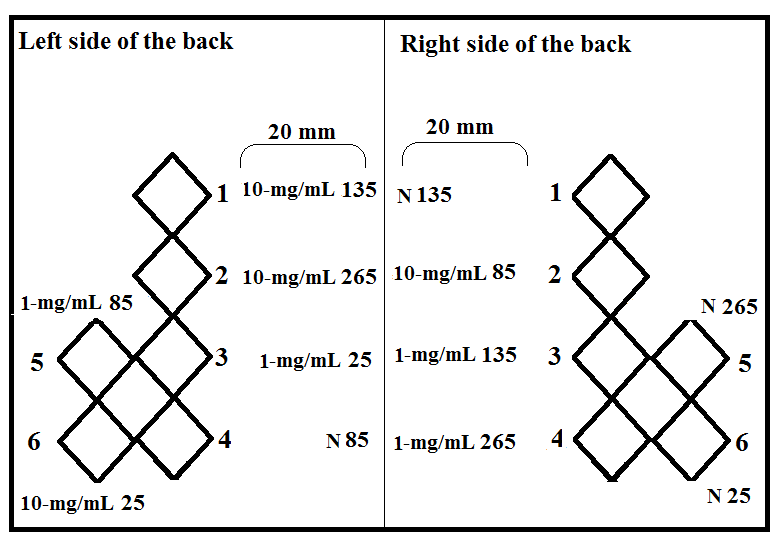 |
