# Supplementary material for: The Lancet Weight Determines Wheal Diameter in Response to Skin Prick Testing with Histamine
Source: PLoS One. 2016 May 23;11(5):e0156211. doi: 10.1371/journal.pone.0156211 (PMC4877047; doi:10.1371/journal.pone.0156211)
Supplement: S1 Table — An outline of various skin prick test guidelines. (DOCX) [file pone.0156211.s002.docx]

## ***Supporting Information S2* – An overview of international skin prick testing guidelines.**

An outline of guideline parameters from Australia, Europe and North America published in position papers [1–3]. These guidelines are routinely revised and updated and the summary below may therefor not reflect the most current clinical practice

|  | **Continents** | | |
| --- | --- | --- | --- |
| **Parameters** | **Australia** | **Europe** | **North America ^*^** |
| Negative control | Mean wheal diameter  < 3 mm | Wheal diameter  < 3 mm | Mean wheal diameter  < 3 mm |
| Positive allergen test | Mean wheal diameter  ≥ 4 mm | Largest wheal diameter  ≥ 3 mm | Mean wheal diameter ≥ 3 mm (above negative control) |
| Positive histamine control | Mean wheal diameter  6 mm | Largest wheal diameter  ≥ 3 mm | Mean wheal diameter ≥ 3 mm (above negative control) |
| Distance between pricks | 20 mm | > 20 mm | 20-25 mm |
| Distance from the wrist | > 50 mm | 20-30 mm | 50 mm |
| Distance from the antecubital fossae | > 30 mm | 20-30 mm | 30 mm |
| Time for reading histamine test results | 10-15 min | 15-20 min | 15 min |
| Time for reading allergen test results | 15-20 min | 15-20 min | 15-20 min |
| Histamine concentration | 10 mg/mL | 10 mg/mL | 10 mg/mL |
| Histamine type | Histamine dihydrochloride | Histamine dihydrochloride | Histamine dihydrochloride |
| ^*^ Recommendations differ for particular types of lancet designs. | | | |

**References:**

1. Berger A: Skin prick testing for the diagnosis of allergic disease. BMJ 2002;325:414.

2. Heinzerling L, Mari A, Bergmann K-C, Bresciani M, Burbach G, Darsow U, et al.: The skin prick test - European standards. Clin Transl Allergy 2013;3:3.

3. Bernstein IL, Li JT, Bernstein DI, Hamilton R, Spector SL, Tan R, et al.: Allergy Diagnostic Testing : An Updated Practice Parameter Allergy Diagnostic Testing : An Updated Practice Parameter. Ann allergy, asthma, Immunol 2008;100.
